# Seed laser priming enhances defensive responses in milk thistle under Pb toxicity

**DOI:** 10.1038/s41598-025-92414-w

**Published:** 2025-03-06

**Authors:** Atefeh Banisharif, Rayhaneh Amooaghaie

**Affiliations:** 1 Plant Science Department, Faculty of Science, Shahrekord University, Shahrekord, Iran; 2 Department of Biology, Faculty of Basic Sciences, Shahed University, Tehran, Iran; 3 Biotechnology Research Institute, Shahrekord University, Shahrekord, Iran

**Keywords:** Antioxidant enzyme, He–Ne laser, Pb tolerance, *Silybum marianum*, Photosynthetic pigments, Proline, Physiology, Plant sciences

## Abstract

Heavy metal stress negatively affects the growth of medicinal plants. While the effects of Helium–Neon (He–Ne) laser on seed germination and stress tolerance in plants has garnered significant attention, little is known concerning the impacts of He–Ne laser irradiation on heavy metal tolerance in plants. Therefore, the current study was conducted to appraise the effect of different durations (0, 20, and 40 min) of seed priming with He–Ne laser (10 mW mm^−2^) on the antioxidant system of *Silybum marianum* L. plants under various Pb concentrations (0, 250, and 500 ppm). Lead phytotoxicity was evident by significant reductions in fresh and dry weights of shoots and roots, total chlorophyll (TChl) content and relative water content (RWC), as well as increases in H_2_O_2_ and malondialdehyde contents in roots and leaves. Seed irradiation with He–Ne laser for 20 min significantly improved these parameters, enhancing Pb tolerance. Conversely, the prolonged laser priming (40 min) resulted in less favorable outcomes, including reduced growth, TChl content, and RWC, while also exacerbating oxidative damage to membranes even under non-stressful conditions. The 20-min laser priming systemically mitigated Pb-induced lipid peroxidation and H_2_O_2_ accumulation by boosting the activities of superoxide dismutase and catalase and increasing proline content in leaves and roots of milk thistle plants. These findings and multivariate analysis suggest that optimal dose of laser initiates a “stress memory” in seeds which is activated upon subsequent exposure to Pb stress, boosting the plant defensive mechanisms and enabling the plant to better cope with oxidative damage. This study underscore the promising potential of He–Ne laser priming as a novel strategy for increasing heavy metal tolerance in medicinal plants like milk thistle, offering an eco-friendly technique for maintaining their productivity under heavy metal stress.

## Introduction

The increasing concentrations of heavy metals in agricultural soils, driven by industrialization, municipal waste production, farming activities, and energy generation, have become a significant environmental concern in many countries^1^. Among these metals, lead (Pb) is recognized as one of the most toxic and has been classified as “a chemical of great concern” under the European REACH regulations^2^. Excessive levels of Pb in soil can disrupt critical physiological processes in plants, including mitosis, photosynthesis, nutrient homeostasis, and water absorption^3^. Additionally, Pb exposure can trigger the generation of reactive oxygen species (ROS) such as singlet oxygen (^1^O_2_), superoxide radicals (O_2_^–^), hydroxyl radicals (OH^–^), and hydrogen peroxide (H_2_O_2_), leading to oxidative stress that damages cellular components like DNA, proteins, lipids, and other essential biomolecules^2,4,5^. These disruptions manifest as toxic symptoms such as leaf chlorosis, wilting of older leaves, and the development of short, brown roots^3^. To mitigate the detrimental effects of heavy metal-induced oxidative stress, plants have evolved antioxidant defense mechanisms. This defense system includes enzymatic antioxidants such as ascorbate peroxidase (APX), peroxidase (POD), superoxide dismutase (SOD), and catalase (CAT), as well as non-enzymatic antioxidants like carotenoids, glutathione, and osmoprotectants such as proline^4,6–10^.

A sharp increase in global heavy metal contamination necessitates the development of innovative strategies to enhance heavy metal tolerance in plants. One promising and cost-effective method is seed priming, which entails pre-germinative treatments using biological, chemical, or biophysical agents to strengthen plants’ adaptive responses under stress conditions^1,9,11–14^. It is believed that the priming agents induce mild stress, leading to epigenetic, transcriptomic, and proteomic modifications that establish a “stress memory” in seeds. This memory enables plants to respond more rapidly and effectively during subsequent exposure to stressful conditions^9,15^. While chemical priming has shown potential to enhance heavy metal stress^4,9,12^, its application might raise concerns regarding potential health risks to humans and environmental safety, particularly in the context of edible and medicinal plants. Therefore, biophysical methods, such as laser-assisted seed priming, have gained significant attention as an eco-friendly and sustainable alternative in recent years^16,17^.

The effectiveness of lasers depends on several factors, including the type of laser used (e.g., helium–neon or diode lasers), wavelength, exposure duration, and seed type^18^. Seed priming with He–Ne laser irradiation has emerged as a novel technique to promote seed germination rate and seedling growth in various plant species under non-stress^17–28^. Laser irradiation is believed to enhance the internal energy levels within seeds, thereby accelerating cell growth and proliferation. The thermal and electromagnetic effects of the laser also stimulate metabolic activities, resulting in improved seed germination and seedling growth^18,27^. Moreover, the wavelength of the He–Ne laser (632.8 nm) corresponds to the absorbance spectrum of the phytochrome and the interaction between laser photons and phytochromes can trigger photochemical effects on biochemical processes^16,29,30^. Recent studies have also explored the efficacy of He–Ne laser seed priming in mitigating various abiotic stresses, including salinity^24^, drought^31^, UV stress^30^ and cadmium sulfide nanoparticle (CdS NPs) toxicity^32^. The transcriptomic analysis demonstrated that He–Ne laser pretreatment enhanced drought tolerance by up-regulating genes associated with photosynthesis, nutrient transport and antioxidant defense in wheat seedlings^33^. Qiu et al.^34^ found that superior performance of laser-primed wheat plants under drought stress was closely associated with their ability to down-regulate several microRNA (miRNA) species and to activate their antioxidant defense mechanisms. However, the effectiveness of the He–Ne laser is species-specific and optimal power and exposure duration are variable for different plants^31,35,36^. It is crucial to note that excessive exposure may have detrimental effects and result in injury to plant tissues^16,37^.

Despite advancements in exploring the beneficial impacts of laser on plants^38^, research on the protective effects of He–Ne laser priming against heavy metal toxicity remains limited, particularly in medicinal herbs. A study showed that exposure to CO_2_ laser radiation (8 min at 20 mW/mm^2^) like magnetic field could increase activities of SOD, CAT, and glutathione reductase, and glutathione (GSH) content in wheat seedlings exposed to Cd and Pb^39^. While there are limited studies concerning the potential benefits of He–Ne laser treatment on cadmium tolerance in wheat^40,41^, its widespread application for other plant species might be limited by challenges such as the lack of standardized protocols. Therefore, further research is required to refine laser parameters suited to medicinal herbs under heavy metal stress. In this regard, the current study investigated the impact of He–Ne laser priming on improving Pb tolerance in milk thistle (*Silybum marianum* L.), an herb recognized for its medicinal properties.

Milk thistle, a member of the Asteraceae family, is a medicinal herb native to the Mediterranean and North African regions^42^. In traditional medicine, the use of *S. marianum* fruits has been recommended to stimulate milk production. Furthermore, milk thistle is prized for its seeds and fruits, which contain flavonolignans collectively known as silymarin. Silymarin exhibits hepatoprotective, antioxidant, anti-inflammatory, antineoplastic, antiviral, antibacterial, and antithrombotic properties^43^. Due to the reduced need for external inputs, this herb often grows in marginal environments^44^ and is increasingly subjected to various environmental stresses, particularly heavy metal contamination. Therefore, enhancing its tolerance to heavy metals is critical for ensuring its production in contaminated soils. To the best of our knowledge, the impact of laser priming on Pb tolerance in *S. marianum* has been not assessed to date. Based on evidence from other plant species, it was hypothesized that He–Ne laser priming can establish a stress memory in milk thistle seeds that can systemically boost the antioxidant defense system in roots and shoots of plants exposed to Pb stress. In this study, we seek to understand how the laser can enhance Pb tolerance in milk thistle and, determine the optimal laser dosage and address applicability challenges to improve Pb tolerance in milk thistle. The findings are expected to provide valuable insights for both researchers and agricultural practitioners, promoting innovative solutions for the sustainable production of medicinal plants in soils contaminated with heavy metals.

## Materials and methods

### Laser exposure, Pb treatment and plant cultivation

This experiment was performed as a factorial arrangement with 3 times of He–Ne laser irradiation (0, 20, and 40 min irradiation) and three Pb concentrations (0, 250, and 500 ppm). Milk thistle (*S. marianum*) seeds were obtained from Pakan Bazr Company in Isfahan, Iran. Surface sterilized seeds were soaked in distilled water before laser irradiation. Then, the surfaces of the seeds were dehydrated and irradiated with a He–Ne laser (beam diameter 12 mm, power density 10 mW mm^−2^, wavelength 632.8 nm). The irradiated seeds were transferred to perlite-containing pots and watered with tap water for one week. Then, seedlings were irrigated with half-strength Hoagland’s solution containing 0, 250, and 500 ppm Pb by adding the required amount of Pb(NO_3_)_2_ salt. Seedlings grown from non-irradiated seeds and untreated with Pb stress were considered as the control.

Plants were grown in a greenhouse under a 12 h photoperiod, light intensity of 400 μmol m^−2^ s^−1^, 25/18 °C day/night temperatures, and relative humidity of 65% for 30 days. Fresh leaves and roots were sampled and immediately frozen in liquid nitrogen and stored at − 80 °C for the various biochemical analyses. In the end, fresh weights of roots and above-ground parts of harvested plants were measured immediately, and dry weights were determined after drying in the oven at 75 °C.

### Measurement of chlorophyll and carotenoids

At first, fresh leaf samples were extracted with 80% pre-chilled acetone and optical absorbance of the filtrate was recorded at 470, 645, and 663 nm by Spectrophotometer. The content of total chlorophyll (TChl.) and carotenoids (Car) was obtained using the following formula^45^:

1 $${\text{TChl}} = \frac{{\left( {20.2{\text{ A}}645 + { }8.02{\text{ A}}663} \right) \times V}}{1000W}$$

2 $${\text{Car}} = \frac{{\left[ {\left( {1000 \times {\text{ A}}470} \right){-}{ }1.82{ }\left( {{\text{Chl}}.{ }a} \right){-}{ }85.02{ }\left( {{\text{Chl}}.{ }b} \right)} \right] \times V}}{{\left( {198{ } \times { }1000{\text{ W}}} \right)}}$$

where D470, D663, and D645 are optical absorbance at 470, 663 and 645 nm, W is the fresh weight of leaf samples, and V is the volume of 80% acetone.

### Measurement of relative water content

The relative water content (RWC) in fresh leaves was determined by the method previously explained by Nabaei et al.^8^ and, the RWC was computed as follows:

3 $$\% {\text{RWC}} = \left[ {\frac{{\left( {{\text{FW}} - {\text{DW}}} \right)}}{{\left( {{\text{TW}} - {\text{DW}}} \right)}}} \right] \times 100$$

where FW, DW and TW are fresh weight, the dry weight (after drying in the oven at 75 °C), and turgor weight of leaf samples (after floating the leaves in water for 14 h) respectively.

### Estimation of malondialdehyde and hydrogen peroxide contents

Heath and Packer^46^ method was adopted to measure malondialdehyde (MDA) content as an indicator of lipid peroxidation. Leaf samples were macerated in trichloroacetic acid and centrifuged for 20 min. The mixture of supernatant (1 ml), thiobarbituric acid, and trichloroacetic acid was incubated and centrifuged. The absorbance of the supernatant was read at wavelengths of 532 and 600 nm, and the MDA content was computed using an extinction coefficient of 155 mM^−1^ cm^−1^ as follows:

4 $${\text{MDA }}\left( {{\text{mM}}} \right) = \frac{{\left( {{\text{A}}532{ } - {\text{ A}}600} \right)}}{155}$$

H_2_O_2_ content was measured by method explained by Anand et al.^47^. The fresh samples were extracted using cold acetone and 1 ml of the resultant filtrate was mixed with 0.2 ml concentrated NH_4_OH solution and 0.1 ml titanium sulfate (5% w/v). Then, this mixture was centrifuged at 10,000×*g* and the resulting sediment was dissolved in 3 ml of 1 M sulfuric acid. The absorbance of the supernatant was read at 415 nm, and H_2_O_2_ content was calculated using the extinction coefficient 0.28 μM cm^−1^.

### Quantification of proline content

The Bates et al. method^48^ was adopted to quantify the proline contents in leaves. Briefly, after macerating leaf samples in 3% sulfosalicylic acid, the homogenate was centrifuged at 4 °C. Then, the mixture of supernatant, glacial acetic acid, and ninhydrin reagent was heated for 40 min. After cooling and adding toluene, the mixture was vortexed and incubated at room temperature. The optical absorbance was recorded at 520 nm, and the proline concentration was estimated using the standard curve.

### Assaying the antioxidant enzyme activities

For enzyme assay, the leaf or root samples (0.1 g) were extracted using 1.5 ml phosphate buffer containing 0.20 g KH_2_PO_4_, 0.14 g Na_2_HPO_4_, 0.8 g NaCl, 0.20 KCl, and 1 g polyvinyl pyrrolidine (PVP) at low temperature and on ice. The extracted tissues were centrifuged (10,000 × g) for 10 min, and the activities of enzymes were assayed in the supernatants according to the method previously explained by Amooaghaie et al.^5^.

SOD activity was assayed by recording a 50% decrease in absorbance at 560 nm, indicating a 50% inhibition in the reduction of nitro blue tetrazolium (NBT) induced by the enzyme. CAT enzyme activity was evaluated based on the decrease in optical absorbance at 240 nm per minute due to the decomposition of H_2_O_2_ and using the extinction coefficient of 39.4 Mm^−1^ cm^−1^.

### Statistical analysis

The experiment was conducted as factorial with a completely randomized design with three replications. For ANOVA analysis, SAS software was applied and, the means were compared by Duncan’s multiple range test at *P* < 0.05. A Pearson’s correlation heat map and principal component analysis (PCA) were generated using R software (version 4.2.2).

## Results

### Impact of He–Ne laser and Pb stress on growth parameters

**Fig. 1:**
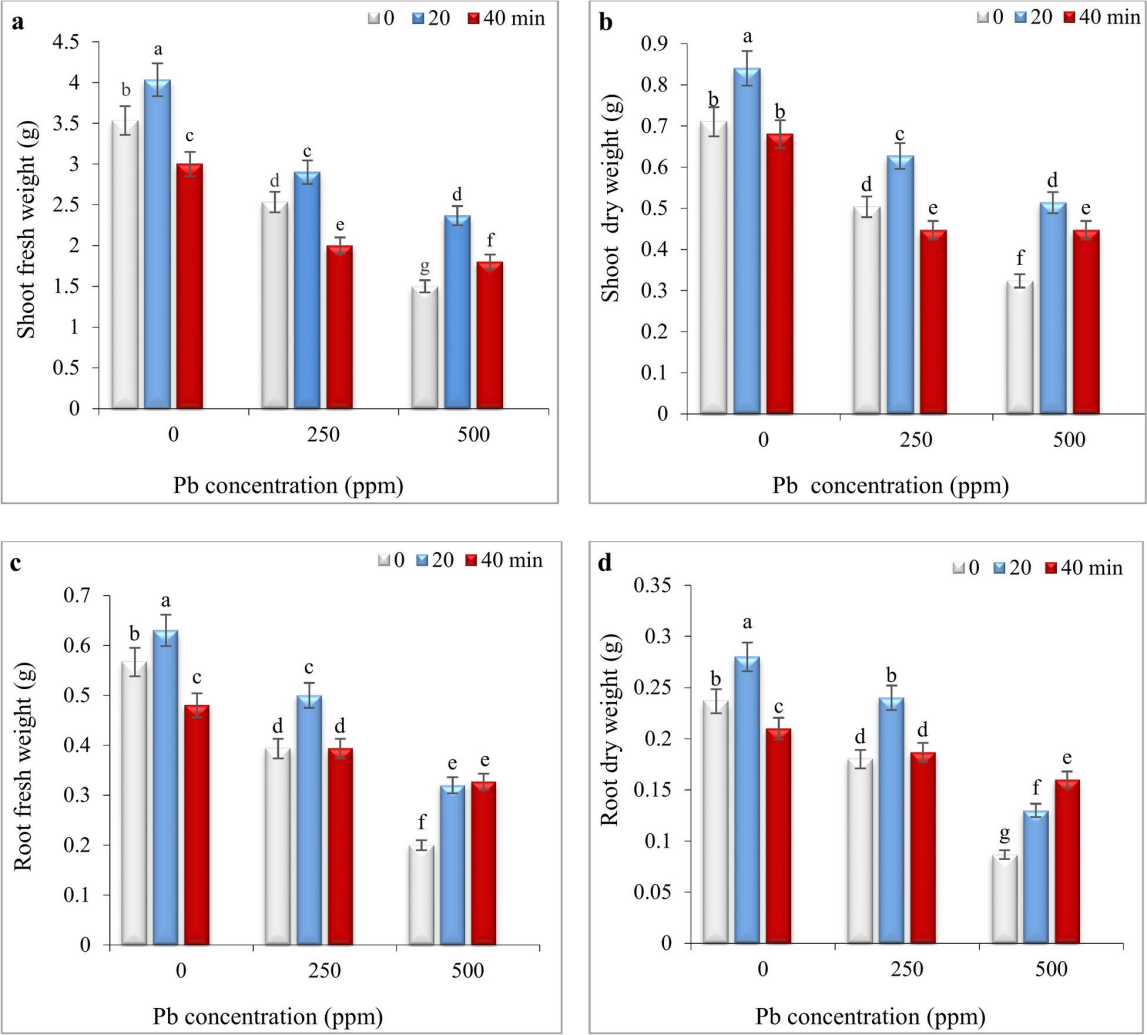
The impact of seed irradiation with He–Ne laser (0, 20, and 40 min) on fresh and dry weights of shoots (**a**,**b**) and roots (**c**,**d**) of *S. marianum* under various Pb concentrations (0, 250 and 500 ppm). Based on Duncan’s multiple-range tests at a significance level of *P* ≤ 0.05, values assigned the same letter indicate that there are no statistically significant differences among them.

Under non-stress conditions, seed laser priming for 20 min significantly increased the fresh and dry weights of shoots and roots. While 40 min laser priming, significantly decreased most growth parameters compared to the control, the dry weight of the aerial parts did not show any meaningful difference from the control (Fig. 1).

Significant decreases in fresh and dry weight of roots and aerial parts were observed at both Pb concentrations (250 and 500 ppm). Seed priming with laser for 20 min significantly alleviated growth inhibition under both Pb concentrations. This treatment increased fresh and dry weight of shoots respectively by 14.62% and 24%, at 250 ppm and by 57.33% and 59.33% at a concentration of 500 ppm Pb (Fig. 1). With increasing radiation time to 40 min, the positive effects of the laser on fresh and dry weight of shoots and roots were reduced at both concentrations of Pb except root dry weight at 500 ppm Pb (Fig. 1).

### Impact of He–Ne laser and Pb stress on photosynthetic pigments

**Fig. 2:**
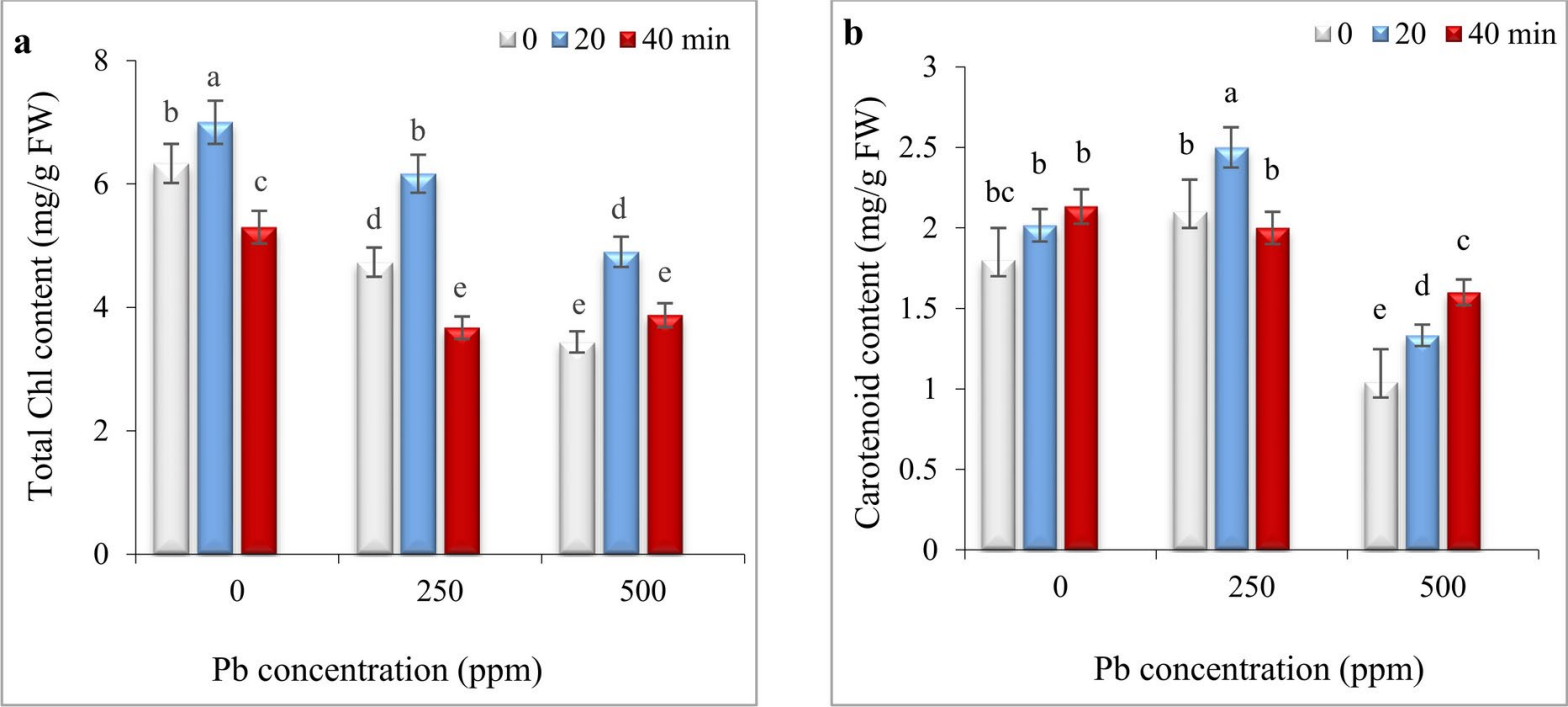
The impact of seed irradiation with He–Ne laser (0, 20, and 40 min) on the content of total chlorophyll (**a**) and carotenoids (**b**) in leaves of *S. marianum* under various Pb concentrations (0, 250, and 500 ppm). Based on Duncan’s multiple-range tests at a significance level of *P* ≤ 0.05, values assigned the same letter indicate that there are no statistically significant differences among them.

While, 20 min laser priming resulted in an enhancement of total chlorophyll (TChl) content by 10.58%, extending the laser priming to 40 min led to a reduction of 16.27% in TChl levels relative to the control under non-stress conditions (Fig. 2a).

Both concentrations of 250 and 500 ppm of Pb significantly reduced TChl contents. He–Ne laser priming for 20 min increased TChl content by 30.35% and 42.44% at 250 and 500 ppm of Pb, respectively, compared to respective controls in these groups. TChl content in plants grown from seeds primed with He–Ne laser for 40 min was equal to or lesser than respective controls at 250 and 500 ppm Pb (Fig. 2a).

Compared to the control, carotenoid content increased by 16.66% at 250 ppm, whereas it reduced by 42.22% at 500 ppm Pb. Seed priming with He–Ne laser for 20 min increased carotenoid content by 19.04% and 27.88% at 250 and 500 ppm, compared to respective controls in these groups. Seed irradiation with He–Ne laser for 40 min did not significantly affect carotenoid content at 250 ppm Pb. Under 500 ppm Pb, carotenoid content in plants grown from seeds irradiated with He–Ne laser for 40 min increased by 53.84% relative to respective control and was higher than in 20 min irradiation in this group (Fig. 2b).

### Impact of He–Ne laser and Pb stress on relative water content

**Fig. 3:**
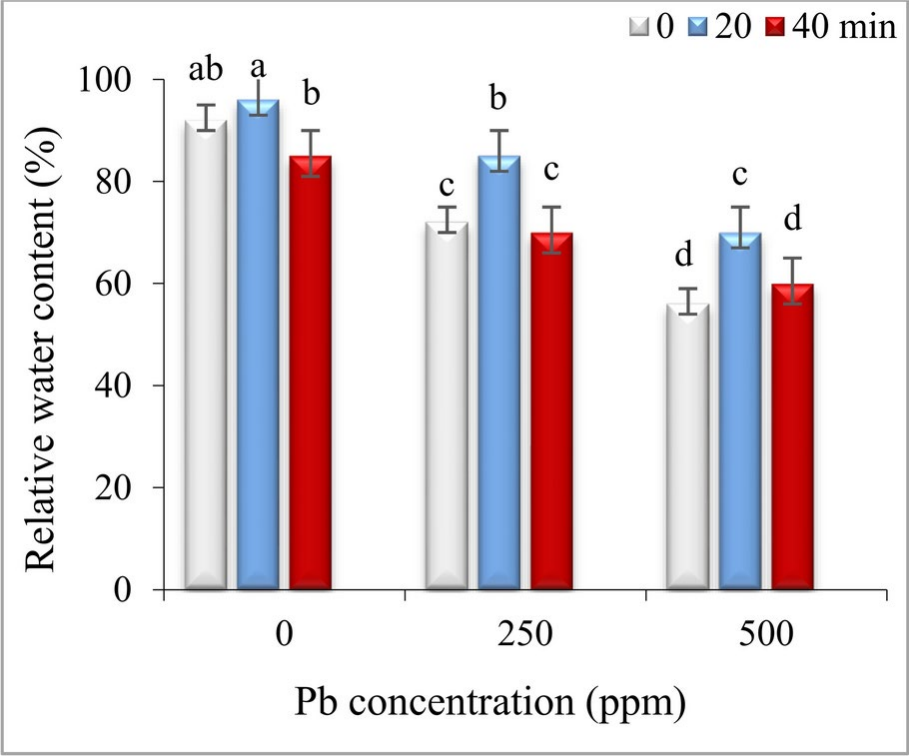
The impact of seed irradiation with He–Ne laser (0, 20, and 40 min) on the relative water content in leaves of *S. marianum* under various Pb concentrations (0, 250, and 500 ppm). Based on Duncan’s multiple-range tests at a significance level of *P* ≤ 0.05, values assigned the same letter indicate that there are no statistically significant differences among them.

Under non-stress conditions, 20- and 40- min laser priming did not result in any significant changes in the relative water content (RWC) of the leaves. Exposure to both Pb levels reduced RWC; however, 20-min laser priming effectively mitigated this reduction. In contrast, 40-min laser priming had no significant effect on RWC compared to the respective controls under 250 and 500 ppm Pb (Fig. 3).

### Impact of He–Ne laser and Pb stress on content of MDA and H_2_O_2_

**Fig. 4:**
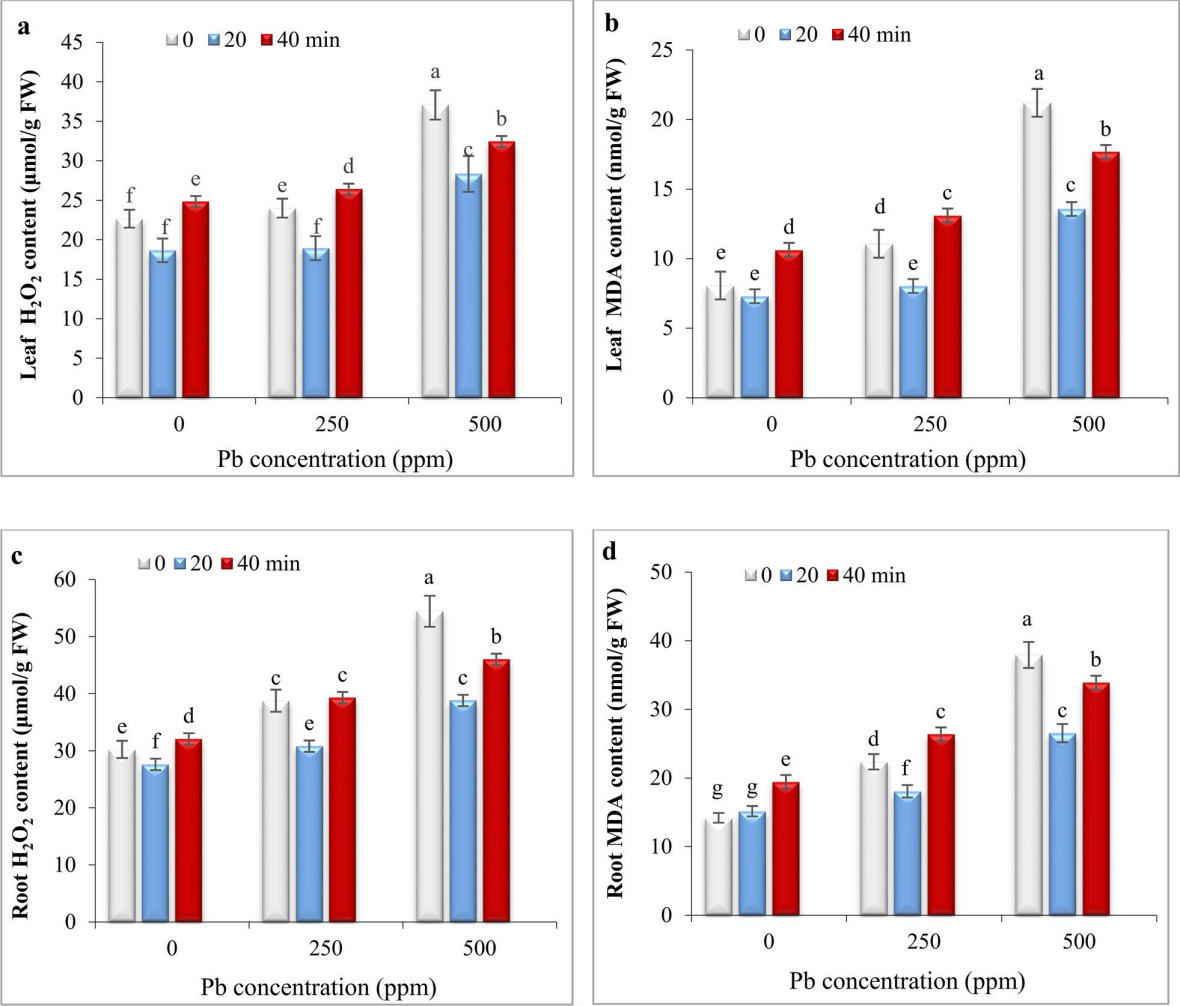
The impact of seed irradiation with He–Ne laser (0, 20, and 40 min) on the content of malondialdehyde (MDA) and hydrogen peroxide in leaves (**a**,**b**) and roots (**c**,**d**) of *S. marianum* under various Pb concentrations (0, 250 and 500 ppm). Based on Duncan’s multiple-range tests at a significance level of P ≤ 0.05, values assigned the same letter indicate that there are no statistically significant differences among them.

Under non-stress conditions, seed priming with He–Ne laser for 20 min did not change the content of H_2_O_2_ and MDA, while 40 min irradiation significantly increased these attributes compared to control in both roots and leaves (Fig. 4 a-d).

The content of MDA and H_2_O_2_ significantly augmented in roots and leaves under both Pb concentrations. The seed priming with He–Ne laser for 20 min significantly decreased the content of MDA and H_2_O_2_ in roots and leaves under both Pb concentrations. In contrast, 40 min laser priming slightly increased the content of MDA and H_2_O_2_ in the roots and leaves of plants exposed to 250 ppm Pb. Under 500 ppm Pb, the content of MDA and H_2_O_2_ in roots and leaves of 40 min primed plants was significantly higher than in the 20 min irradiated groups but was lower than values in non-irradiated plants (Fig. 4).

### Impact of He–Ne laser and Pb stress on proline content

**Fig. 5:**
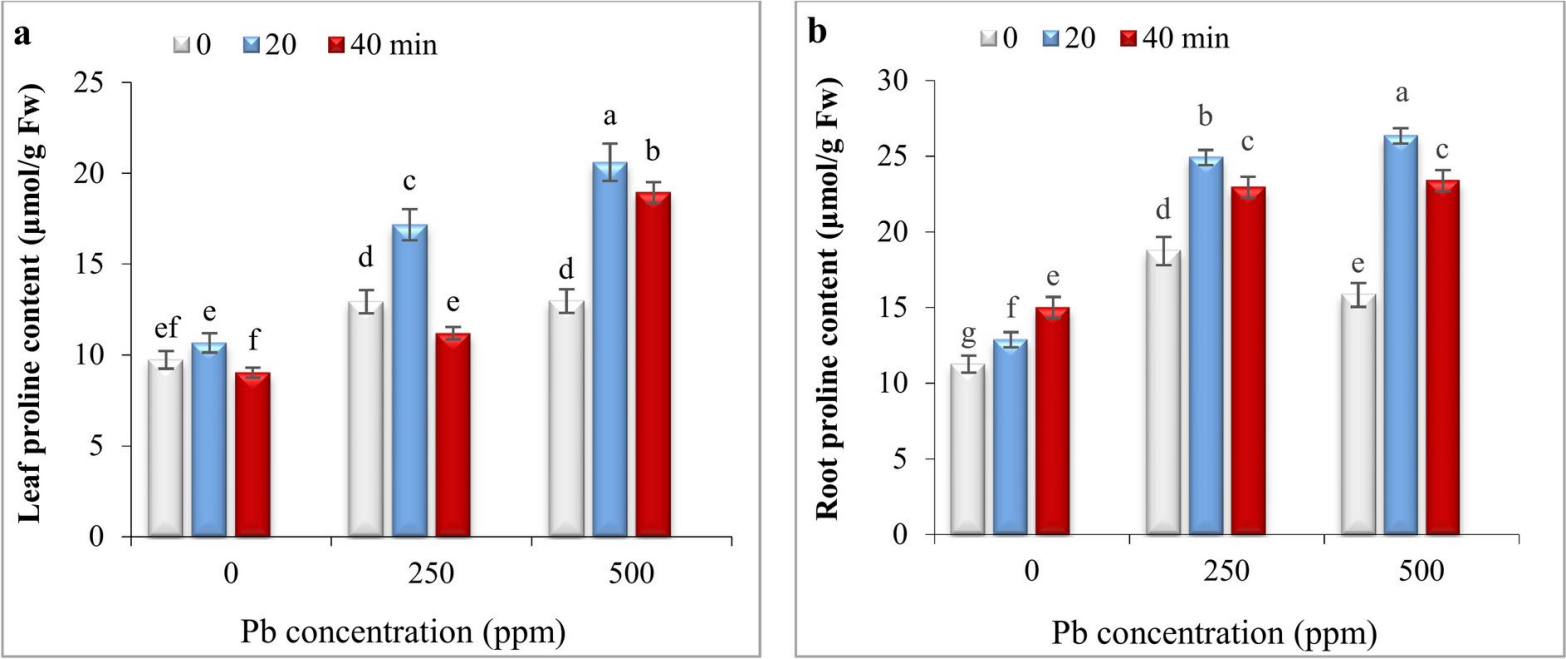
The impact of seed irradiation with He–Ne laser (0, 20, and 40 min) on proline content in leaves (**a**) and roots (**b**) of *S. marianum* under various Pb concentrations (0, 250, and 500 ppm). Based on Duncan’s multiple-range tests at a significance level of *P* ≤ 0.05, values assigned the same letter indicate that there are no statistically significant differences among them.

While 20- and 40-min laser priming did not change proline content in leaves; these treatments significantly increased it in roots under non-stress conditions (Fig. 5).

Our results showed that proline content in leaves and roots significantly increased under 250 and 500 ppm Pb. While 20- and 40-min laser priming enhanced proline content in leaves and roots compared to respective controls under both Pb concentrations, proline content in leaves of 40- min- primed plants Pb was lower than respective control under 250 ppm. Overall, proline content in leaves and roots was significantly lower at 20-min than 40-min-primed plants under both Pb levels (Fig. 5).

### Impact of He–Ne laser and Pb stress on the activity of antioxidant enzymes

**Fig. 6:**
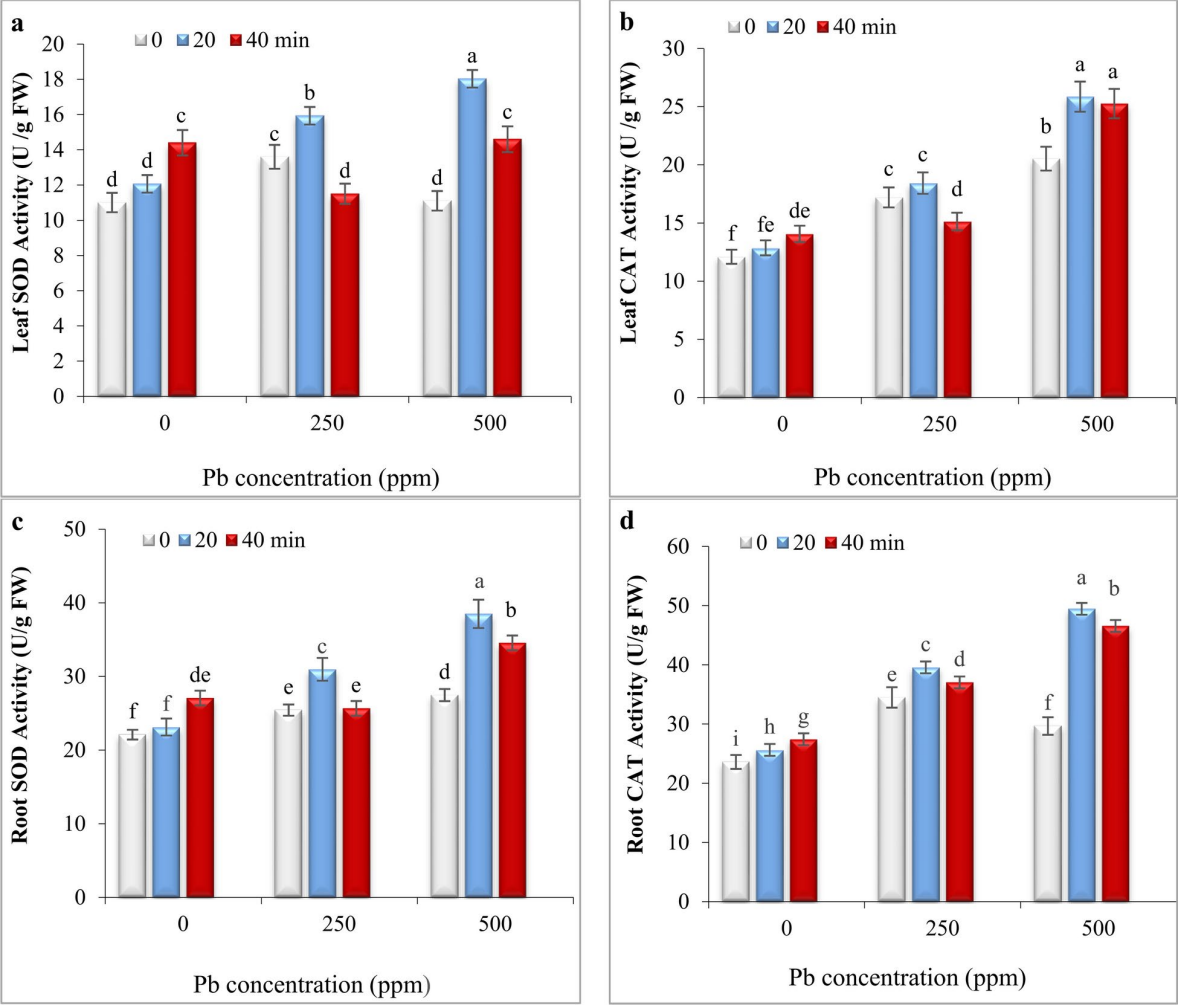
The impact of seed irradiation with He–Ne laser (0, 20, and 40 min) on the activity of SOD and CAT enzymes in leaves (**a**,**b**) and roots (**c**,**d**) of *S. marianum* under various Pb concentrations (0, 250, and 500 ppm). Based on Duncan’s multiple-range tests at a significance level of *P* ≤ 0.05, values assigned the same letter indicate that there are no statistically significant differences among them.

The effect of laser priming on the activity of SOD and CAT in leaves and roots of *S. marianum* has been shown in Fig. 6.

Under normal conditions, seed irradiation with He–Ne laser for 20 min did not change the SOD and CAT activity in leaves and SOD activity in roots but slightly increased CAT activity in roots. Seed irradiation with He–Ne laser for 40 min increased deramatically the SOD and CAT activity in leaves and roots under normal conditions (Fig. 6). The activity of SOD and CAT in leaves and roots significantly increased at 250 ppm Pb. However, the SOD activity in leaves had no significant difference with control under 500 ppm Pb. The 20-min laser priming enhanced the activity of both enzymes in roots and leaves under 250 and 500 ppm Pb (Fig. 6).

Under 250 ppm Pb, the enzymatic activities in leaves and roots of 40 min laser-irradiated plants were less than those in 20 min laser-irradiated plants as well as were often less or equal to control (except CAT in the root). Under 500 ppm Pb, the enzymatic activities in leaves and roots of 40 min laser-irradiated plants were less or equal to 20 min laser-irradiated plants. Still, they were significantly higher than values in non-primed plants (Fig. 6).

### Multivariate analysis

**Fig. 7:**
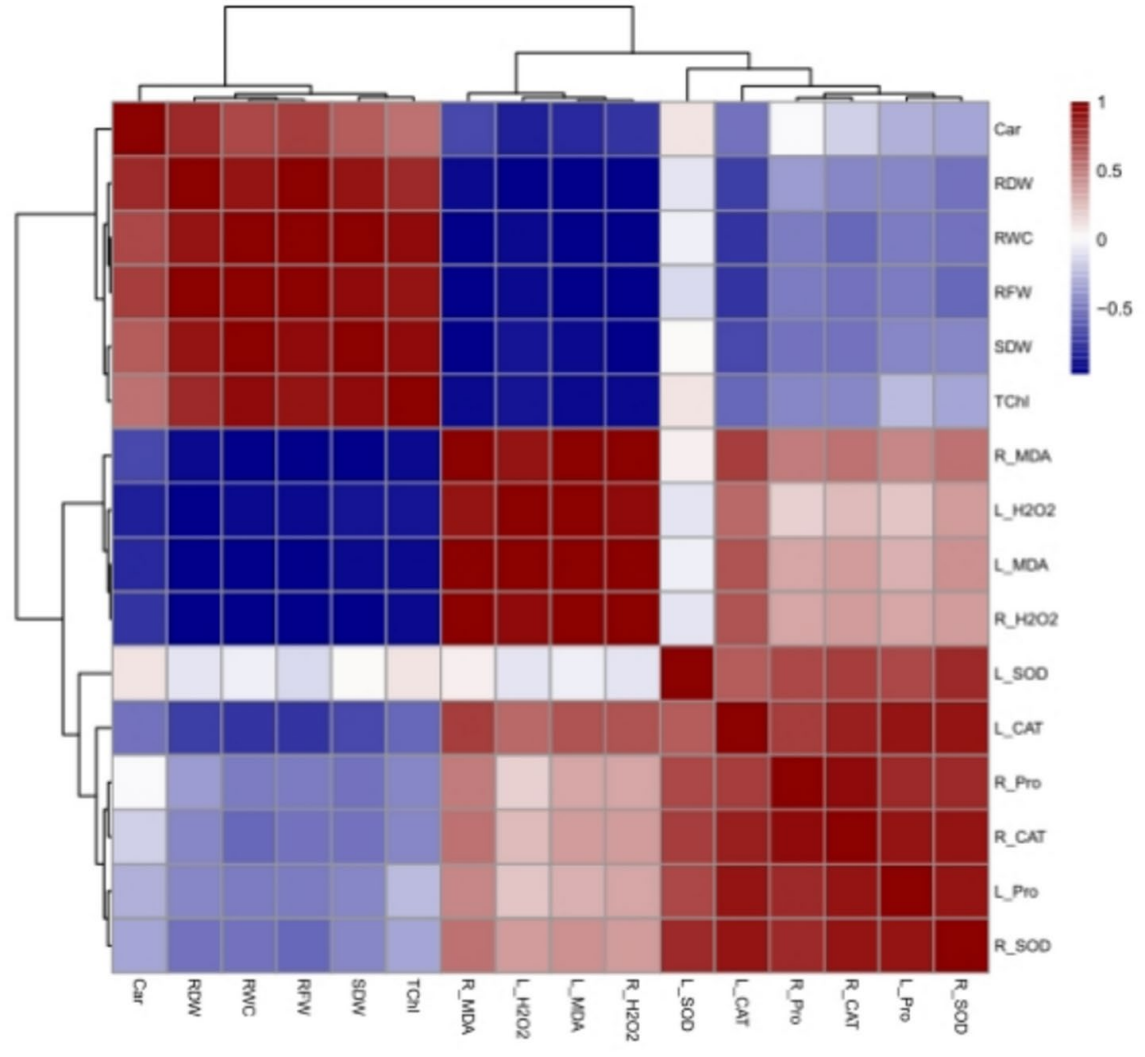
Heat map based on Pearson’s correlation coefficient correlations between all variables under various levels of Pb stress and laser priming in the leaves and roots of *S. marianum*. Strong positive and negative correlations were depicted by dark red and dark blue colors, respectively. Please see the abbreviation of variables in the capture of Fig. 8

The heat map derived from Pearson’s correlation analysis (Fig. 7) revealed a negative correlation between oxidative biomarkers (H_2_O_2_ and MDA contents) in leaves and roots, and several parameters such as the fresh and dry weights of shoots and roots, total chlorophyll content, and relative water content (RWC). Furthermore, the Pearson’s correlation results demonstrated a negative correlation between plant growth attributes and the activities of antioxidant enzymes as well as proline content in roots and leaves (Fig. 7).

**Fig. 8:**
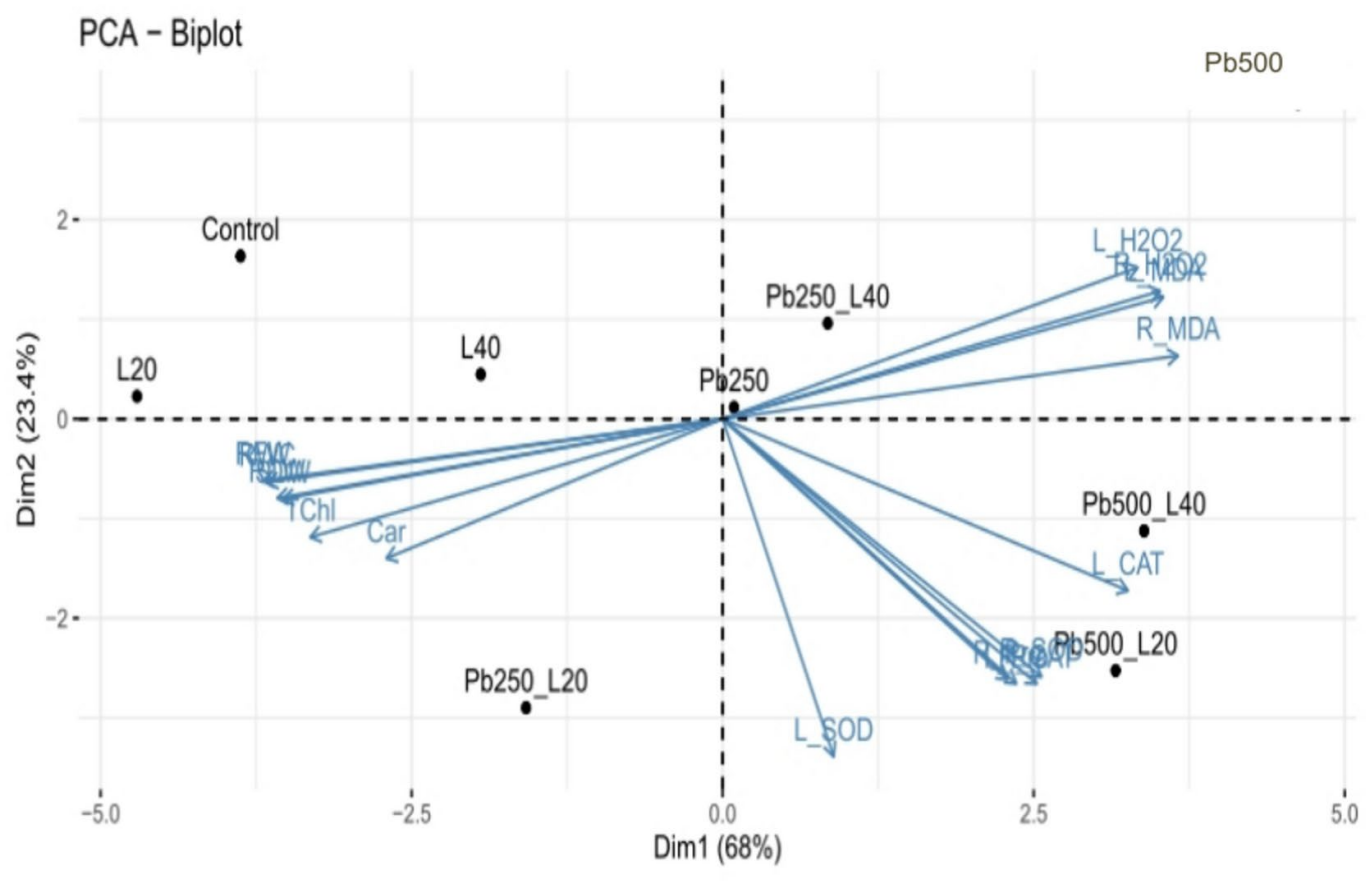
Association between the different groups of variables and treatments via PCA. The investigated variables are shoot and root fresh weight (SFW, RFW), shoot and root dry weight (SDW, RDW), relative water content (RWC), total chlorophyll content (TChl), carotenoid content (Car), leaf and root malondialdehyde (L-MDA, R-MDA), leaf and root hydrogen peroxide (L-H_2_O_2_, R-H_2_O_2_), leaf and root proline content (L-Pro, R-Pro), leaf and root superoxide dismutase activity (L-SOD, R-SOD), leaf and root catalase activity (L-CAT, R-CAT) in *S. marianum*. The treatments were included: control, seed priming with 20 and 40 min He–Ne laser (L20, L40), Pb 250 ppm + 20 min He–Ne laser (Pb250-L20), Pb 250 ppm + 40 min He–Ne laser (Pb250-L40), Pb 500 ppm + 20 min He–Ne laser (Pb500-L20) and Pb 500 ppm + 40 min He–Ne laser (Pb500-L40).

The PCA showed the association between the various groups of treatments and variables. The PCA components showed PCA 1 (68%) and PCA 2 (23.4%) data variability. As shown in Fig. 8 most the increasing H_2_O_2_ and MDA contents in leaves and roots are associated with Pb500, Pb500 + L40, and Pb250 + L40 treatments. In the second group, the variables of L-CAT, L-SOD, R-CAT, R-SOD, L-Pro, and RPro are associated with Pb500 + L20 treatment. In the third group, TChl, Car*,* growth parameters, and RWC are associated with L20 and Pb250 + L20 treatments (Fig. 8).

## Discussion

As the challenge of heavy metal pollution increasingly threatens plant productivity in worldwide, it is essential to develop innovative solutions to enhance plant tolerance. In this regard, the present study explored the effects of Pb stress on *S. marianum* and the potential of He–Ne laser priming as a mitigation strategy.

Lead toxicity negatively impacts the growth and development of plants by interfering with essential cellular functions including respiration, photosynthesis, water balance, and cell division^2^. In the current study, Pb stress significantly reduced growth parameters (Fig. 1) and TChl content (Fig. 2a) in *S. marianum*, which was consistence with previous reports on milk thistle^49^, quinoa^4^, and sesame^5^. Seed priming with He–Ne laser irradiation for 20 min significantly alleviated these detrimental effects of Pb toxicity, offering its potential to enhance heavy metal tolerance in medicinal plants. Zhu et al.^41^ also reported that He–Ne laser priming can effectively mitigate cadmium toxicity in wheat by modulating nutrient uptake and enhancing antioxidative defense mechanisms. The stimulatory effects of He–Ne lasers are ascribed to mechanisms such as converting absorbed light energy into chemical energy^17^, and the interaction between laser photons and phytochromes^29,50^. Experimental evidence revealed that phytochromes play a crucial role in laser-induced defensive responses^16,29,30^. Phytochromes are photoreceptors that regulate various physiological processes, including seed germination, flowering, growth, chloroplast development, Chl biosynthesis and stress tolerance^51,52^. A study showed that seed priming with He**–**Ne laser increased level of *phy B* transcripts in leaves that was closely linked to improved growth parameters and salt tolerance of *Salvia officinalis* seedlings^16^. Therefore, the role of phytochrome in initiating “stress memory” in milk thistle seeds and subsequent regulation of growth, Chl biosynthesis and defensive system in Pb-exposed plants is possible. Future studies should validate the accuracy of this hypothesis using *phy-*deficient mutants. Importantly, extending the laser exposure to 40 min diminished the positive effects of laser priming on milk thistle plants, suggesting that the duration of laser treatment is critical for achieving optimal outcomes. Similar trend was observed in triticale, where seed laser irradiation for 3 h resulted in increased seedling length, fresh and dry weights, and chlorophyll content in leaves. Conversely, extending the irradiation duration to 24 h led to a reduction in these attributes^53^. Aslam et al.^37^ also reported that 2 min He–Ne laser irradiation enhanced plant length, leaf number, and fresh and dry weight of roots and shoots in drought-stressed wheat, while higher doses (5 min exposure) had inhibitory effects. Likely, the variability in effective doses among different plant species depends on factors such as structure, anatomy, and inherent susceptibility of seeds to light and laser power and exposure method^50^.

The negative effect of Pb on growth parameters was strongly correlated to decreasing chlorophyll content (Fig. 2a) and disrupting water balance (Fig. 3). The reduction in chlorophyll content can be due to the inhibition of enzymes involved in chlorophyll biosynthesis or may be attributed to the oxidative damage of chlorophyll by ROS generated by Pb toxicity^2^. The 20-min laser priming preserved chlorophyll content (Fig. 2b) and maintained safeguarding turgor required for cellular growth (Fig. 3), improving photosynthesis efficiency and the plant’s ability to sustain growth and biomass of milk thistle under both Pb concentrations. Likewise, seed irradiation with He–Ne laser improved the content of chlorophyll *a, b,* and carotenoids in leaves of salt-stressed *Salvia officinalis*^15^ and increased RWC in drought-stressed wheat^34^. The biosynthesis of chlorophyll is regulated by light^54^. The laser also is a monochromatic light that can induce biosynthesis of photosynthetic pigments, increasing photosynthetic efficiency in plants^24,35,53^. It is also known that laser application can up-regulate key photosynthesis-related genes, including those responsible for encoding PS-II, chlorophyll-binding protein, and ATP synthase. This mechanism likely contributes to enhanced photosynthetic efficiency and overall plant growth^33^. Herein, laser priming reduced ROS generation by increasing carotenoid content (Fig. 2b) and enhancing antioxidant enzyme activity (Fig. 6), thereby decreasing oxidative degradation of chlorophyll. Carotenoids play a protective role by preventing the diversion of surplus electrons toward oxygen, thereby reducing ROS formation and chlorophyll degradation^5,9,55^. Laser-primed plants exhibit more extensive root systems, which enhance water uptake efficiency, particularly in Pb-contaminated soils. The observed rise in RWC in the leaves of Pb-exposed milk thistle plants may also be attributed to the elevated accumulation of proline (Fig. 5). Proline plays a critical role in regulating osmotic potential and preserving cellular turgor under diverse stress conditions, including heavy metal stress^8,56^. Similarly, seed laser irradiation increased proline content and improved the growth of water deficit-exposed sunflowers^35^ and salt-stressed *Salvia officinalis*^57^. The positive correlation between RWC and TChl with growth parameters, (Fig. 7) highlights the critical role of RWC and photosynthetic pigments in enhancing the biomass of milk thistle plants. PCA results (Fig. 8) also showed that this effect was more significant in plants subjected to 20-min laser priming particularly under non-stress conditions and in the presence of 250 ppm Pb. However, extending the radiation time to 40 min diminished the positive effects of the laser treatment. Similarly, 2 min laser priming increased chlorophyll *a, b,* total chlorophyll and carotenoid contents, improved photosynthetic efficiency, growth parameters and final yield in wheat, whereas these attributes decreased under 5 min laser exposure^37^.

In the current study, Pb-induced oxidative stress was evident from increased levels of H_2_O_2_ and malondialdehyde (MDA) in leaves and roots of *S. marianum* (Fig. 4 a-d) and, 20 min laser priming could alleviate it under both Pb concentrations. Previous studies also exhibited that laser exposure reduced ROS contents (H_2_O_2_ and O_2_^·−^) in leaves of tall fescue plants exposed to CdS nanoparticles^32^ and, diminished MDA production in leaves of drought-stressed wheat plants^34^ and salt-subjected *Withania somnifera* (L.)^36^ by activating antioxidant system. The findings of the current study also demonstrated a positive correlation between H_2_O_2_ and MDA levels with proline content and the activity of antioxidant enzymes (Fig. 7). Furthermore, the PCA results (Fig. 8) revealed a close association of defensive responses with the Pb250 + L20 and Pb500 + L20 treatments. Proline not only maintains the water balance but also can ameliorate heavy metal-induced oxidative stress via its function as a scavenger of ROS and a metal chelator^7,8^. The observed increase in proline levels (Fig. 5) might be related to laser-induced transcriptional memory related to proline metabolism. The light-dependent proline accumulation has been earlier reported in salt-stressed Arabidopsis^58^. Feng et al.^59^ showed light priming led to more substantial proline accumulation in rice plants under subsequent salt exposure. They demonstrated that light-responsive transcription factors can establish a memory of proline biosynthesis genes, enabling faster and stronger proline accumulation during subsequent stress exposures. The laser priming for 20 min also systematically boosted the activities of SOD and CAT enzymes in both roots and leaves (Fig. 6). These enzymes play a crucial role in scavenging ROS and mitigating oxidative damage^4,7–9^. The stimulatory effect of laser on antioxidant enzymes might be related to the induction of signals such as nitric oxide and H_2_O_2_^32,57^ and high transcription of respective genes^33^. Moreover, the magnetic effects of laser irradiation may interact with metal ions in enzyme structures (e.g., Mn^2^⁺, Zn^2^⁺, Fe^2^⁺), modulating their activity and further enhancing ROS scavenging capacity^60^. Qiu et al.^40^ also found that seed priming with He–Ne laser for 5 min decreased MDA, H_2_O_2_, and O_2_^·−^ contents by up-regulating the transcription levels and the activities of CAT, POD, APX, SOD and increasing content of AsA and GSH in Cd-exposed wheat seedlings.

The study indicates that a 20-min laser exposure improved Pb tolerance in milk thistle, whereas a 40-min exposure compromised the antioxidant defense mechanisms in both roots and leaves, as shown in Fig. 6. This variation may be attributed to the differing levels of H_2_O_2_ produced in seeds under low and high laser dosages. A previous study also demonstrated that an optimal dose of He–Ne laser enhanced salt tolerance in *Salvia officinalis* by boosting a mild and transient H_2_O_2_ burst^16^. It is known that the lower levels of H_2_O_2_ as a signal molecule confer a protective effect, whereas high concentrations of H_2_O_2_ cause oxidative stress under various stresses^61^. Likely, optical, electromagnetic, and thermal effects of laser^17^ in plants subjected to 20-min laser priming motivated a controlled and transient ROS burst, resulting in a “stress memory” creation. This stress imprint is subsequently remembered in Pb-exposed seedlings, enabling rapid and robust activation of defense mechanisms and sustaining Chl content, photosynthesis and biomass of plants. In contrast, 40-min laser priming likely generated excessive light intensity, severe magnetic fields, or high heat, leading to the overproduction of ROS in seeds. This resulted in heightened oxidative stress and an accelerated rate of lipid peroxidation, which may have surpassed the capacity of the plant’s antioxidant defense systems. This conclusion was further supported by PCA results that revealed most contents of H_2_O_2_ and MDA in leaves and roots were associated with treatments involving 40-min laser priming under Pb stress (Pb500 + L40 and Pb250 + L40). The antioxidant system in these plants subsided compared to 20 min laser primed-plants and was insufficient to counteract the heightened oxidative stress, resulting in compromised growth and physiological performance under both non-stress and Pb stress conditions.

## Conclusion

The findings of this study highlight the potential of He–Ne laser seed priming as a cost-effective and environmentally friendly approach to mitigate Pb toxicity in medicinal plants. The results suggest the brief stress induced by laser irradiation establishes a “stress memory” in seeds and primes the plants for future challenges such as Pb toxicity. This memory systematically boosts antioxidant enzyme activity and proline accumulation in roots and leaves and improves plant performance under Pb stress. Further research should elucidate the molecular mechanisms underlying laser-induced “stress memory”, such as alteration of histones, transcription factors, signaling molecules, and phytochromes. This study also underscores the importance of optimizing laser dosage to achieve the desired outcomes. Therefore, further research is needed to establish species-specific guidelines for laser priming. Overall, the optimal dose of laser represents a simple, non-destructive, and eco-friendly technology with long-term benefits. Despite its promising potential, most studies on laser have been conducted under controlled laboratory conditions. To fully realize the potential of laser priming in advancing sustainable agriculture, extensive large-scale field trials and robust standardization efforts are essential.

## Data Availability

The data that support the findings of this study are available from Atefeh Banisharif (Email: atefehbanisharif@gmail.com) upon reasonable request.

## Declarations

### Permission to collect samples

The permission to sowing *Silybum marianum* L. was acquired from Agricultural and Natural Resources Ministry of Iran.

### Statement on experimental research and field studies on plants

The plants *Silybum marianum* L. sampled comply with relevant institutional, national, and international guidelines and domestic legislation of Iran.
